# Isolation of a lytic bacteriophage against extensively drug‐resistant *Acinetobacter baumannii* infections and its dramatic effect in rat model of burn infection

**DOI:** 10.1002/jcla.24497

**Published:** 2022-06-16

**Authors:** Ehsanollah Ghaznavi‐Rad, Majid Komijani, Alireza Moradabadi, Marzieh Rezaei, Nima Shaykh‐Baygloo

**Affiliations:** ^1^ Department of Medical Laboratory Science Faculty of Paramedicine Arak University of Medical Sciences Arak Iran; ^2^ Molecular Research Center Faculty of Medicine Arak University of medical sciences Arak Iran; ^3^ Department of Biology Faculty of Science Arak University Arak Iran; ^4^ Molecular and medicine research center Khomein University of Medical Sciences Khomein Iran; ^5^ Department of Cell, Molecular Biology and Microbiology Faculty of Biological Sciences and Biotechnology University of Isfahan Isfahan Iran; ^6^ Department of Biology Faculty of Science Urmia University Urmia Iran

**Keywords:** *Acinetobacter baumannii*, antibiotic resistance, bacteriophage, burn, phage therapy

## Abstract

**Objectives:**

*Acinetobacter Baumannii* is an opportunistic nosocomial pathogen belonging to the *Moraxellaceae* family. The emergence of multidrug resistant strains of this pathogen caused many problems for hospitals and patients. The aim of the current study was to isolate, identify, and morphologically, physiologically, and in vivo analyze a new lytic bacteriophage targeting extensively drug‐resistant (XDR) *A. baumannii*.

**Materials and Methods:**

Different wastewater samples were tested for isolation of lytic bacteriophage against 19 *A. baumannii* isolates obtained from patients hospitalized in a hospital in Arak, Iran, from January 2019 to March 2019. The phenotypic and genotypic characteristics of *A. baumannii* strains (resistance genes including: *adeA*, *adeB*, *adeC*, *adeR*, *adeS*, *ISAba1*, *blaOXA‐23*, *blaOXA‐24*) were analyzed. The isolated phage characteristics including adsorption time, pH and thermal stability, host range, one‐step growth rate, electron microscopy examination, and therapeutic efficacy of the phage were also investigated. Therapeutic efficacy of the phage was evaluated in a rat model with burn infection of XDR *A. baumannii.* The lesion image was taken on different days after burning and infection induction and was compared with phage untreated lesions.

**Results:**

The results showed unique characteristics of the isolated phage (vB‐AbauM‐Arak1) including high specificity for *Acinetobacter baumannii*, stability at a relatively wide range of temperatures and pH values, short adsorption time, short latent period, and large burst size. In relation to the therapeutic efficacy of the phage, the lesion area decreased in phage‐treated groups over 14 days than in those untreated, significantly (*p* < 0.05).

**Conclusion:**

Our findings demonstrated that isolated lytic phage was able to eliminate burn infections caused by XDR *A. baumannii* in a rat model. So, it may be recommended as alternative options toward to developing a treatment for extensively drug resistant *Acinetobacter* infections.

## Graphical abstract

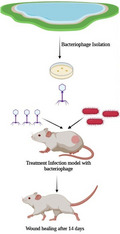

Antibiotic resistance is a serious global problem. One of the most effective ways to treat infections is to use bacteriophage (phage therapy). The present study tested a bacteriophage isolated from wastewater to treat burn infection caused by XDR *Acinetobacter Baumannii* in rats. The results showed that the application of bacteriophages for treatment was effective in improving burn infection

## 1 INTRODUCTION

*Acinetobacter baumannii* is an encapsulated non‐motile aerobic gram‐negative coccobacillus. As a multidrug resistant (MDR) pathogen, it causes opportunistic infections including pneumonia, meningitis, urinary tract infections, sepsis, and wound and burn infections.^1^, ^2^ Recently, the resistance rate of *A. baumannii* was obviously increased, and the resulting infections are considered a critical threat.^3^, ^4^ Many reports implied that the mortality rate is yearly increasing all over the world due to high levels of antibiotic resistance.^5^, ^6^ Regarding clinical findings, and World Health Organization (WHO) report, while it is the post‐antibiotic era, infections caused by antibiotic‐resistant bacteria could lead to an increase in the mortality rate, the treatment cost, and hospitalization time.^7^, ^8^ According to Stanton et al.^9^ report, if immediate and effective actions are not taken against antimicrobial resistance, more than 10 million deaths will occur worldwide by 2050, with a huge financial burden. Indeed, about 700,000 people die each year due to these infections. WHO priority pathogens list for Research and Development (R&D) of new antibiotics is showed that the *A. baumannii*, carbapenem‐resistant‐associated infections are critical. New antibiotics will help to reduce deaths from resistant infections worldwide by targeting the pathogens on this list.^10^

Nowadays, researchers are focusing on bacterial antibiotic resistance as a global problem and choosing the best alternative in solving it. The bacteriophages could be that alternatives way. Bacteriophages are viruses that only infect bacteria without any effect on the eukaryotic cells. It means they have specific hosts. They are reproducible and do not have any side effects.^11^ Increased susceptibility of the burn surfaces to the rapid colonization of multiple bacterial species or strains could lead to mixed infection and the formation of bacterial biofilms with resistance to multiple antibiotics.^11^, ^12^ Therefore, the simultaneous use of bacteriophage and topical antibiotics could be applied to treat mixed infections that occurred on burn surfaces.^13^, ^14^ Although conventional topical antibiotics are widely used, they have significant disadvantages, including antibiotic resistance. Thus, there is an urgent need for new therapeutic approach to control infections caused by the MDR‐ and extensively drug resistant (XDR) pathogens.

In numerous studies, lytic bacteriophages with lysis potential of the Extended Spectrum Beta‐Lactamase (ESBL), MDR, XDR, and PDR bacterial strains have been isolated.^11^, ^15^, ^16^, ^17^, ^18^ Nowadays, the use of bacteriophage as an alternative antibacterial drug against various infectious diseases has been reported. Phage therapy would be suggested as a promising antibacterial alternative, in cases where current antibiotics cannot control infections.^19^, ^20^, ^21^, ^22^ On the other hand, previous studies in food and water treatment, biocontrol, and care have shown that the future applications of bacteriophages will be clear and promising.^23^, ^24^ So far, numerous lytic phages have been isolated against MDR *A. baumannii*, which have been used in successful clinical trials.^25^ In relation to the treatment of burn infections, the efficacy of lytic phages has been proven in several studies.^26^, ^27^, ^28^ However, in the study of Jault et al.^26^ on burn infections caused by *Pseudomonas aeruginosa*, phage cocktail efficacy was at a slower rate than the standard of care (1% sulfadiazine silver emulsion cream) due to the rapid drop of phage titers, and low susceptibility of *P. aeruginosa* to the phage cocktail that should be considered in future studies.

Thus, further detailed investigations including gene sequencing, bacterial susceptibility to the phage, host range analysis, in vivo examinations, and treatment limitations will help further approval of bacteriophage application.

This study reports the isolation of the potent lytic phage from wastewater sources against XDR infections of *A. baumannii* that may be considered a candidate for phage therapy.

## 2 MATERIAL AND METHODS

### 2.1 Sample collection

Experimental procedures were approved by the Ethical Committee of Arak University of Medical Sciences (IR.ARAKMU.REC.1398.019). Nineteen clinical isolates of XDR *A. baumannii* were isolated from patients admitted to Valiasr Hospital of Arak from January 2019 to March 2019. Identification of all the isolated bacteria were confirmed by using biochemical tests (Gram staining, indole, methyl red, Voges Prausker, citrate, triple sugar iron agar, oxidative/fermentative, urease test, coagulase test, catalase, and oxidase test for confirmation).^29^ Before further analysis, informed consent has been obtained.

### 2.2 DNA Extraction

The bacterial genomic DNA was extracted using the boiling method. At first, colonies from pure isolated bacteria were placed into a tube containing 100 μl of DNase and RNase free distilled water (DENA Zist Asia, Iran), boiled at 100°C for 12 min. The cells were then pelleted by centrifugation, and the supernatant containing DNA was stored at −20°C until use.^30^

### 2.3 Molecular detection

Identification of *A. baumannii* isolates was confirmed by specific Polymerase Chain Reaction (PCR) and then sequencing of PCR products. PCR was done using primers Ac696F (5´‐TAYCGYAAAGAYTTGAAAGAAG‐3′) and Ac1093R (5´‐CMACACCYTTGTTMCCRTGA‐3′) designed for partial sequence of Zone 1 of RNA polymerase β‐subunit (*rpoB*) gene. DNA samples from 19 isolates were amplified in a total volume of 25 μl, containing 3 μl of template DNA, 0.4 μM of each primer, 0.2 mM of each dNTP, 50 mM KCl, 20 mM Tris–HCl, 2 mM MgCl2, 1 U Smar *Taq* DNA polymerase (CinnaGen, Iran), and sterile deionized water. The PCR products (about 397 bp) were then analyzed by agarose gel electrophoresis (Sigma‐Aldrich, USA). The sequencing of PCR products was performed by Macrogen Company (Republic of Korea). Finally, sequences were recorded in GenBank and the phylogenetic tree was constructed by MEGA software version X.^26^, ^31^

### 2.4 Phenotypic and genotypic resistance pattern of bacteria

The genotypic resistance patterns of 19 *A. baumannii* isolates were investigated by PCR using specific primers (Table 1). The materials used in PCR are the same as those mentioned in section “Molecular detection” and the PCR conditions were set according to Table 1.

**TABLE 1:** Primers used to detect genotypic resistance patterns

Gene	Sequence (5^/^ to 3^/^)	Product size (bp)	Annealing temperature (°C)	References
*adeA*	F:ATCTTCCTGCACGTGTACAT R:GGCGTTCATACTCACTAACC	513	56	^32^
*adeB*	F:TTAACGATAGCGTTGTAACC R:TGAGCAGACAATGGAATAGT	541	56	^32^
*adeC*	F:AGCCTGCAATTACATCTCAT R:TGGCACTTCACTATCAATAC	560	56	^32^
*adeR*	F:ACTACGATATTGGCGACATT R:GCGTCAGATTAAGCAAGATT	447	52	^32^
*adeS*	F:TTGGTTAGCCACTGTTATCT R:AGTGGACGTTAGGTCAAGTT	544	52	^32^
*ISAba1*	F:CATTGGCATTAAACTGAGGAGAAA R:TTGGAAATGGGGAAAACGAA	451	52	^33^
*blaOXA‐23*	F:GATCGGATTGGAGAACCAGA R:ATTTCTGACCGCATTTCCAT	501	53	^34^
*blaOXA‐24*	F:GGTTAGTTGGCCCCCTTAAA R:AGTTGAGCGAAAAGGGGATT	249	54	^34^

Susceptibilities of the bacterial isolates to 19 different antimicrobial agents (Mast,UK) (Cefoxitin, Cefotaxime, Ceftazidime (30 μg), Cefepime, Piperacillin, Piperacillin/Tazobactam (100/10 μg), Amoxicillin/Clavulanic acid, Aztreonam, Ciprofloxacin (5 μg), Levofloxacin, Erythromycin, Clindamaycin, Chlorafenicol, Rifampin, Merpenem, Gentamicin (10 μg), Amikacin (10 μg), Trimethoprim/Sulfamethoxazole (1.25/23.75 μg) and Colisitin (10 μg)) were defined by using the standard disk diffusion method according to Clinical and Laboratory Standard Institute (CLSI).^35^ All meropenem‐resistant isolates were confirmed by Epsilometer (E test) and carbapenemase inhibition method tests. The diameters of the zones of inhibition around the discs, after overnight incubation at 37°C, were recorded and compared with the interpretive criteria recommended in the CLSI guidelines.^35^

### 2.5 Phage isolation

In order to isolate and enrich the bacteriophage against *A. baumannii*, a standard protocol of phage isolation was carried out with some modifications. Several urban wastewaters (Arak, Iran) samples were collected and subjected to centrifugation (5000 g, 10 min). Then, the supernatant was filtrated through a 0.45 μm sterilized syringe filter. One milliliter of an overnight culture of *A. baumannii* was added to 30 ml of 2× brain–heart infusion (BHI) broth. After 4 h incubating (37°C, with constant shaking at 100 g), 30 ml of the filtered wastewater was added to the medium and incubated overnight under the same condition. Then, the suspension was subjected to centrifugation as mentioned above and the obtained supernatant was filtrated (0.45 μm).^36^ Afterward, the spot test technique was used to investigate the presence of a specific lytic phage in the suspension. The appearance of the transparent zone (plaque) was considered as the existence of *A. baumannii* lytic bacteriophage. 

Bacteriophage purification was performed according to the double‐layer overlay method using serial dilution (10^−1^ – 10^−12^ pfu/ml) of the phage suspension in a mixture of Sodium chloride, Magnesium sulfate, and gelatin (SM buffer). Finally, a single plaque was selected and then, the propagation of phage was carried out routinely according to Sambrook and Russell protocol.^37^ The procedure of phage purification was repeated three successive times.

### 2.6 Characterization of phage morphology by electron microscopy

The purified phage particles were subjected to transmission electron microscopy for characterizing the morphological features. A very high titer (approximately 10^10^ pfu/ml) was acquired by centrifugation (60,000 g, 45 min) and was added to the surface of a carbon‐coated copper grid. Then, after 2 min, the uracil acetate 2% (w/v) (Sigma‐Aldrich, USA) was used for negative staining. The stained grids were air‐dried and were visualized by transmission electron microscopy (Philips, Netherlands) at an accelerating voltage of 100 kV. After identifying the phage characteristic based on the morphology of the head and tail, the name and the family of the phage were determined according to the Kropinski method.^38^

### 2.7 Phage adsorption rate

To determine adsorption rate, the phage suspension (10^8^) was mixed with overnight culture of *A. baumannii* (multiplicity of infection or MOI of 0.01) and incubated at 37°C. Samples (100 μl) were taken after 3, 5, 8, 10, 15, and 20 min and diluted in 850 μl SM buffer and 50 μl chloroform. After centrifugation (8000 g, 2 min) at 4°C, the supernatants were titrated for determination of unadsorbed phages by the double‐layer method.^39^, ^40^ All process has been repeated three times.

### 2.8 Host range

The host range of the phage was assessed using both standard strains and clinical isolates of a number of gram‐positive and gram‐negative bacteria (Table 2). Standard spot assay was used to study the sensitivity of the bacteria; which presence of a clear plaque was considered positive.

**TABLE 2:** Determination of host range: +, Clear plaque; −, no plaque

Bacterial species	Description	Activity
*Acinetobacter baumannii*(19 isolates)	XDR isolates	+ (12 isolates)
*Pseudomonas aeruginosa*	ATCC 27853	−
*Pseudomonas aeruginosa*	Burn infection	−
*Escherichia coli*	ATCC 25922	−
*Escherichia coli*	Urinary tract infection	−
*Proteus mirabilis*	MDR isolates	−
*Klebsiella pneumonia*	MDR isolates	−
*Shigella sonnei*	Wastewater	−
*Shigella flexneri*	Wastewater	−
*Aeromonas hydrophila*	ATCC 7965	−
*Bacillus cereus*	PTCC 1247	−
*Streptococcus pneumonia*	ATCC 49619	−
*Enterococcus faecalis*	ATCC 29212	−
*Staphylococcus aureus*	ATCC 35933	−
*Staphylococcus saprophyticus*	ATCC 15305	−

### 2.9 Thermal and pH stability

For the temperature stability test, the phage suspension (10^8^) was incubated at 4, 25, 40, 50, 60, 70 and 80°C, and then phage titer was measured after 1 h incubation using a double‐layer agar plate method.^41^ For the pH stability test, phage titer (10^8^) was determined after 1 h incubation of phage lysates mixed with equal volume of pH buffer solutions of the different range of pH values (pH 2, 3, 4, 5, 6, 7, 8, 9, 10, 11, 12, and 13). The control group of pH and thermal stability experiments were pH of 7 and a temperature of 25°C, respectively.^42^ All process has been repeated three times.

### 2.10 One‐step growth assay and burst size

To determine the latent period (the interval between adsorption of the phage to the bacterial cell and release of phage progeny) and burst size (the ratio of the final count of released phage particles to the count of infected bacterial cell during latent period) of the phage, one‐step growth experiment was performed. Burst size and latent period were investigated using analysis of one‐step growth curve. For one‐step growth experiment, a volume of 20 ml overnight culture of *A. baumannii* (OD600 of 0.3) was centrifuged (6000 g, 5 min). The pelleted cells were resuspended in 0.5 ml brain–heart broth (37°C). The phage suspension (10^8^) was added to the bacterial suspension at MOI of 0.01 and allowed to adsorb for 10 min at 37°C. The mixture was centrifuged (10,000 g, 1 min) to remove unadsorbed phages. The infected bacterial cells were resuspended in 10 ml brain–heart broth (37°C) and incubated at 37°C. Samples were taken at 5‐min intervals up to 40 min and 10‐min intervals up to 80 min, and immediately titrated.^40^, ^43^

### 2.11 Experimental animals and burn infection wound

To evaluate the in vivo efficacy of the bacteriophage, twenty male Wistar rats weighing 200–300 g (age between 5–8 weeks) were purchased from the animal facility center of Arak University of Medical Sciences. They were kept in individual cages maintained at 20–26°C and 40–70% humidity, with a standard light cycle. The cage and water bottle were sterile in the autoclave before they were used in the study. Animals were fed with standard rodent dried food, provided with water ad libitum. For animal anesthesia, intraperitoneal injections of 50 mg/kg ketamine and 20 mg/kg xylazine using G25 syringes were done. All procedures were carried out in accordance with the National Institutes of Health Animal Care Guidelines.

Twenty male rats were used in this study (to avoid the gender difference effect), which were randomly divided into 4 groups with five rats per group: group 1, infected animals without treatment; group 2, infected animals treated with phage (10^8^); group 3, infected animals treated with glycine as phage diluent; group 4, animals without infection as self‐healing control.

### 2.12 Burning model

Skin burns were created as described by Durmus et al. and Hosnuter et al.^44^, ^45^ The third‐degree burn lesion on the back of the animal was made as follows: We used a 1‐cm‐diameter metal rod, it was placed in boiling water for 40 seconds to become 100°C. After that, it was put on the back of the animal for 30 seconds. Before burning the anesthetized animal, an electric shaver was used to expose the skin surface.

### 2.13 Infection model and wound closure analysis

The inoculation of *A. baumannii* was performed on the same day on which the burning lesion was made. XDR *A. baumannii* was inoculated onto the burned wound at 0.5 McFarland standard solution. Phage therapy was done approximately 10 hours after the inoculation of *A. baumannii*. The lesion image was taken in different days (1, 7, 14, and) after burning and infection induction. Finally, after treatment, the presence of bacteria in the lesions was examined by culturing. The lesion areas were measured in mm^2^ using the NIH ImageJ software (https://imagej.nih.gov/ij/download.html) and compared with each other. The infiltration and cell aggregation in the non‐treatment and the phage‐treated groups were compared after Hematoxylin and eosin (H&E) staining (400×). The lesion after 14 days punch out and the skin was prepared for H&E staining. Preparation of the H&E staining has been done as follows: at first, skin burn samples from each rat were fixed in 10% neutral‐buffered formalin. The fixed specimens were embedded in paraffin, sectioned at 3–4 μm thickness, stained with Hematoxylin solution for 20–40 min, decolorized with 70% ethanol‐containing 1% HCl‐for 5 seconds, stained in Eosin solution for 10 min and dehydrated, cleared, mounted and finally viewed under a microscope. All samples were washed between each step of H&E staining, for 1–5 min in tap water. Scoring of skin burn lesions in all groups were compared on the basis of the most characteristic features observed in the skin burns.^46^

### 2.14 Statistical analysis

GraphPad Prism software version 6.1 (GraphPad Software Inc., USA) and One‐way ANOVA were used to analyze the results. *P* values of <0.05 were considered as statistically significant.

## 3 RESULTS

**FIGURE 1:**
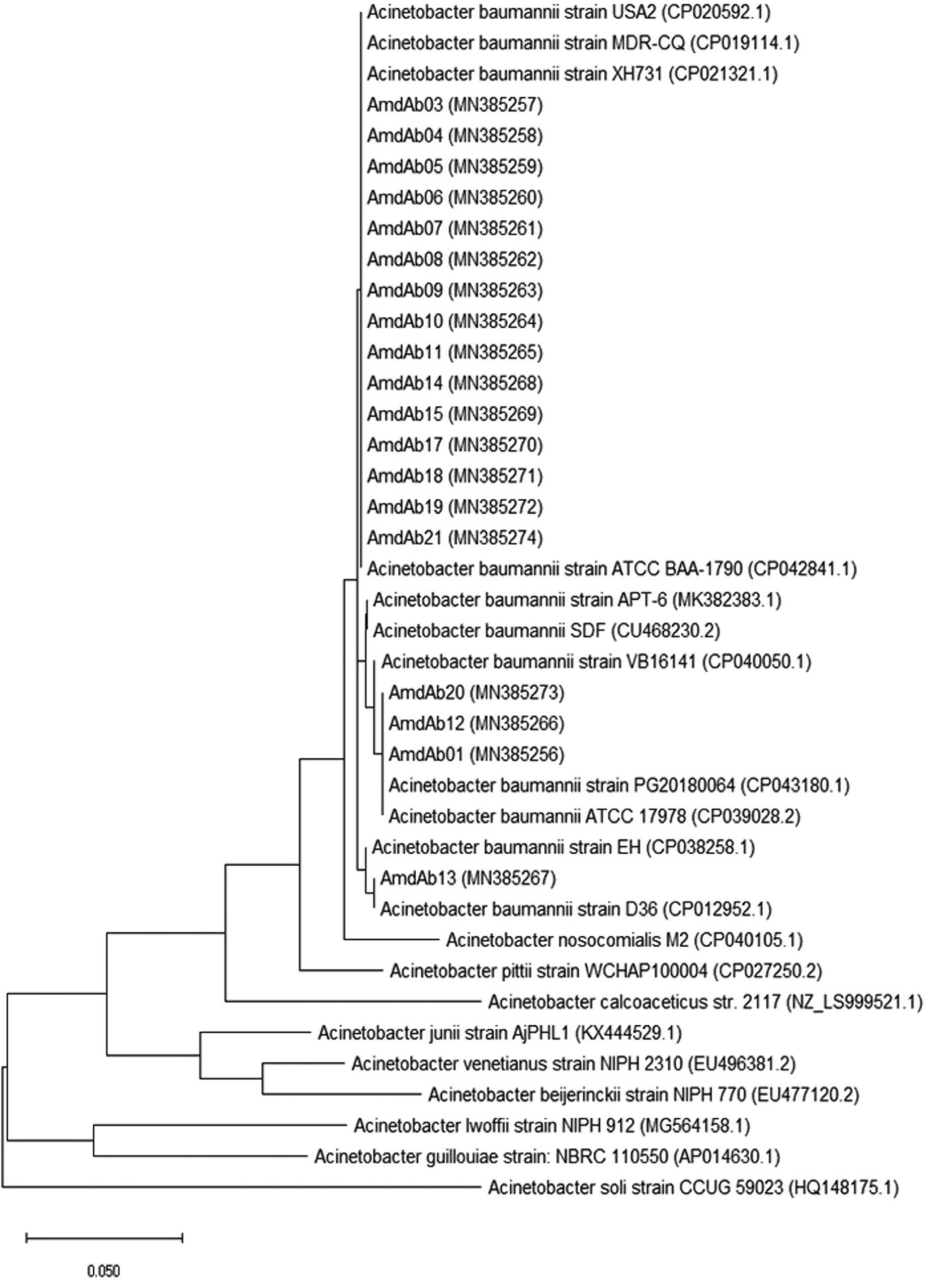
The Phylogenic tree of Maximum Likelihood *Acinetobacter*

The sequencing results of PCR products of 19 isolates are available on the GenBank nucleotide database under accession numbers MN385256 to MN385274. Sequences analysis using Basic Local Alignment Search Tool (BLAST) was shown that all 19 isolates belong to *A. baumannii* species. The Maximum Likelihood phylogenetic tree of *Acinetobacter* strains containing 19 isolates obtained in this study was shown in Figure 1.

### 3.1 Phenotypic and genotypic pattern of antibiotic resistance

All of the isolates were resistant to all tested antibiotics, except Colistin. Among the 19 bacterial isolates, 9 isolates were positive for *blaOXA*‐24 gene (47%), 18 isolates were positive for *adeC* and *adeS* genes (94.7%), and 17 isolates were positive for *adeR* gene (89.4%). All of the isolates (100%) were positive for *blaOXA*‐23, Insertion sequence ISAba1, *adeA*, and *adeB* genes.

### 3.2 Characterization of phage morphology by electron microscopy

**FIGURE 2:**
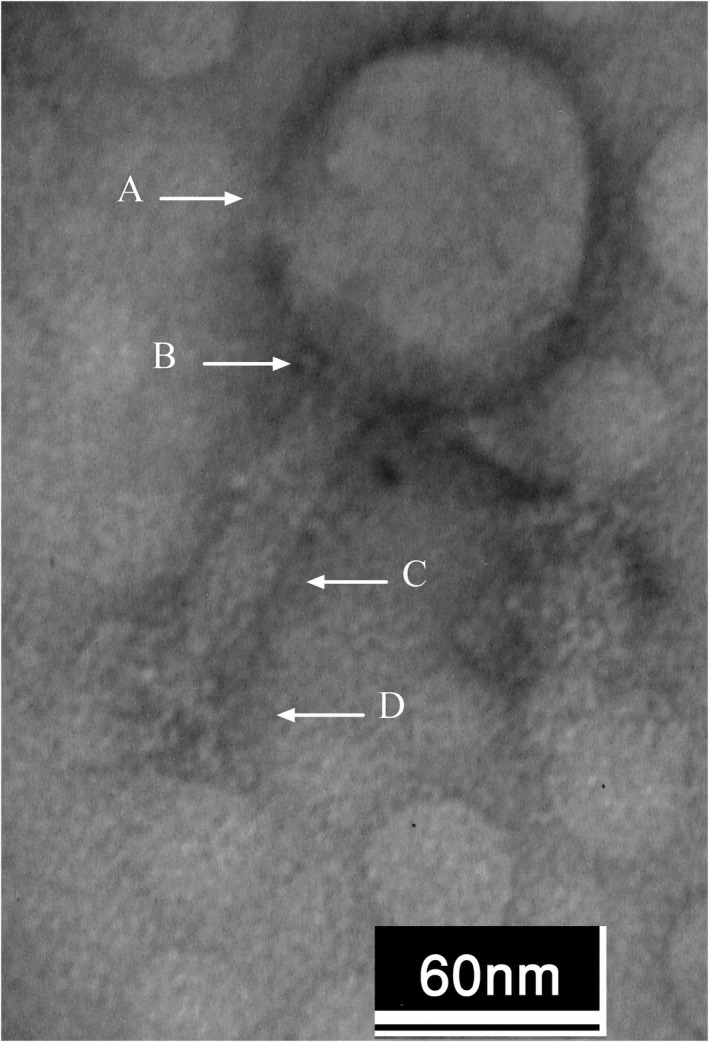
Characterization of phage Morphology by Electron Microscopy, A: Head, B: Neck, C: Tail, D: Endplate

The isolated phage had a head with a width of 90 ± 2 nm and a length of 101 ± 3 nm, a tail with a length of 103 ± 2, and endplates with a length of 36 ± 2 nm. The morphological characteristics investigated by electron microscopy (Figure 2), indicated that the isolated phage (vB‐AbauM‐Arak1) belonged to the *Myoviridae* family.

### 3.3 Host range

According to the spot assay results, 12 isolates from 19 isolates (63%) of *A. baumannii* were lysed by vB‐AbauM‐Arak1 and no clear plaques were observed in other tested bacteria (Table 2). The host range determination results showed that phage vB‐AbauM‐Arak1 was specific to *A. baumannii*.

### 3.4 Determination of Phage Stability at Different pH and Temperature Values

**FIGURE 3:**
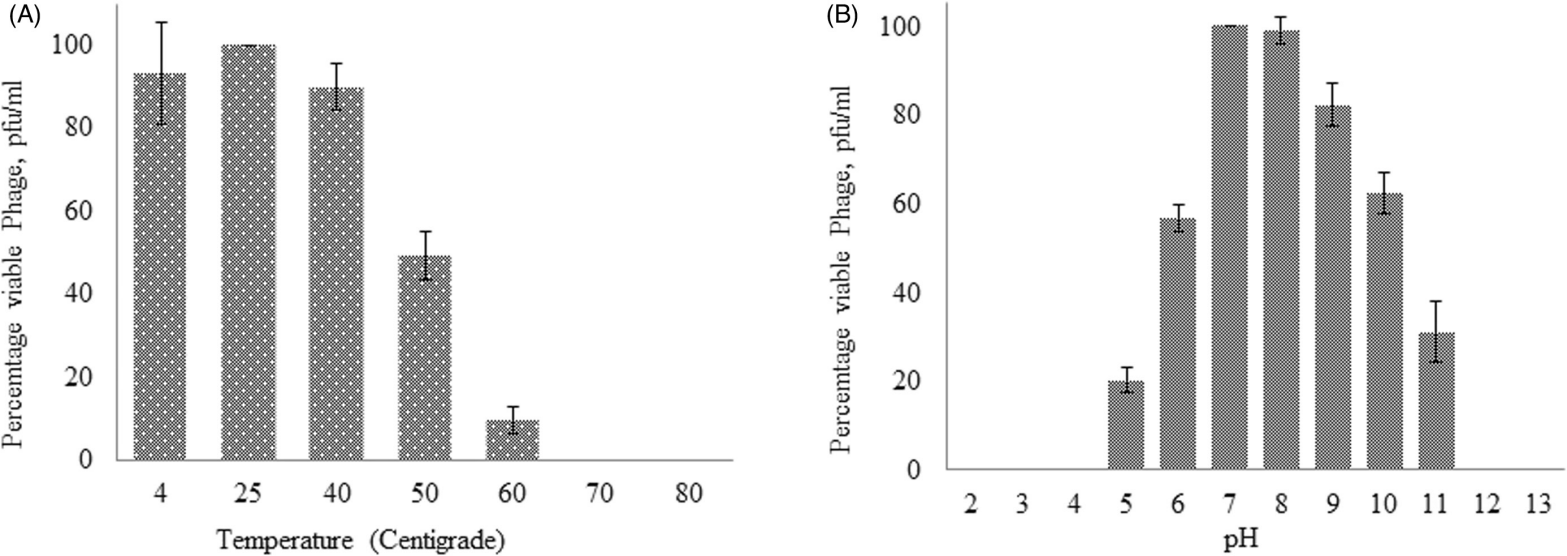
Stability of the phage vB‐AbauM‐Arak1 at (A) different temperatures and (B) pH values

The vB‐AbauM‐Arak1 phage was fully inactivated only at 70 and 80°C. The optimal temperature of the phage lysis activity against *A. baumannii* infection was confirmed with the observation of a completely transparent zone of inhibition. As shown in Figure 3A, The 90%, 100%, 85%, 50%, and about 10% percentages of phage titer were preserved after 1 h of incubation at 4, 25, 40, 50, and 60°C, respectively. The phage titer was dramatically reduced to almost zero at 60°C and the phage was completely inactivated at 70 and 80°C (Figure 3A).

The stability of the phage at different pH values (2–13, and) was evaluated after determining the titer of the active phage by the overlay method. The result is shown in Figure 3B. At the pH of 7 and 8, the viable phage percentage pfu/ml was measured at about 100% and more than 90%, respectively. However, the phage titer reduced at other pH values.^5^, ^6^, ^9^, ^10^, ^11^ The activity was ultimately stopped at pHs 2, 3, 4, and 13 (Figure 3B).

### 3.5 Phage adsorption rate and plaque size

**FIGURE 4:**
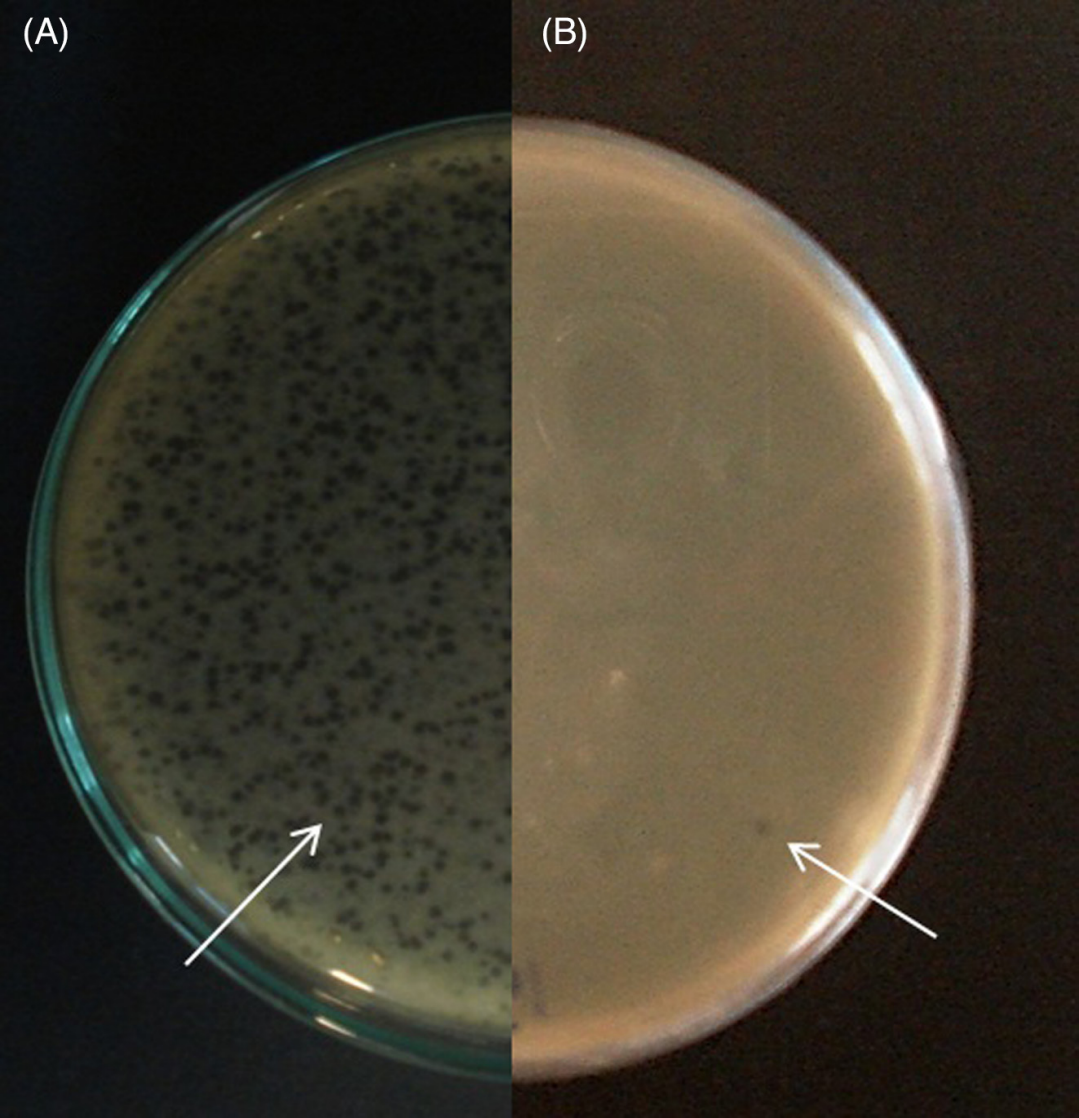
(A) Bacteriophage plaques before purification (B) Individual plaque used for purification

The results showed, 99% of the phage particles adsorbed on the bacterial cells within 10 min after incubation of the mixture of the phage and host bacterial cells at 37°C. Although the size of the individual plaque was approximately 2 mm (Figure 4).

### 3.6 Latent period and phage burst size

**FIGURE 5:**
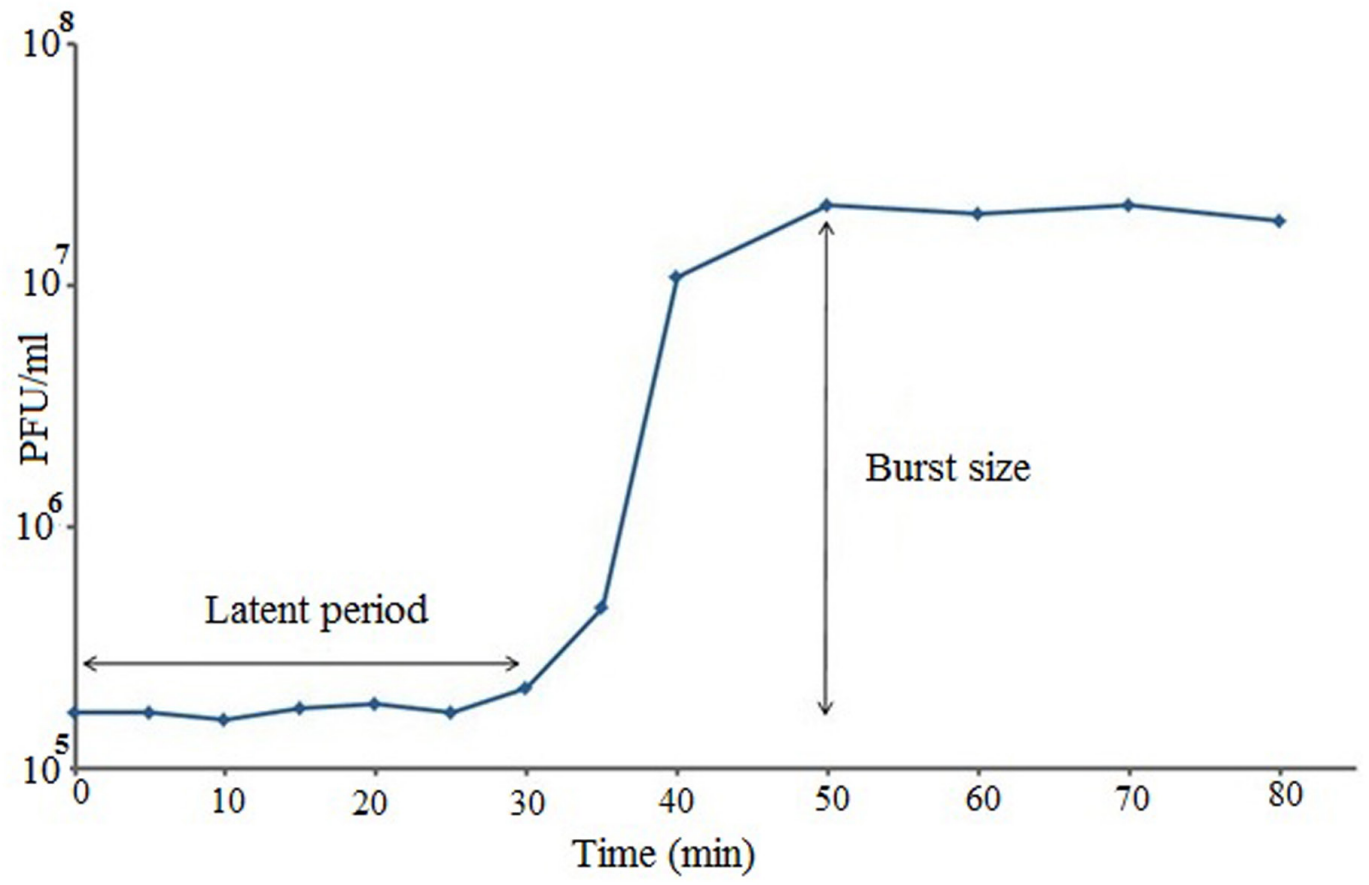
One‐step growth curve. The phage has a latent period of about 30 min. The burst size was approximately 200 phage particles per infected cell

As shown in Figure 5, the latent period of the phage was approximately 30 min and the burst size was about 200 phage particles per one infected cell.

### 3.7 Wound area

**FIGURE 6:**
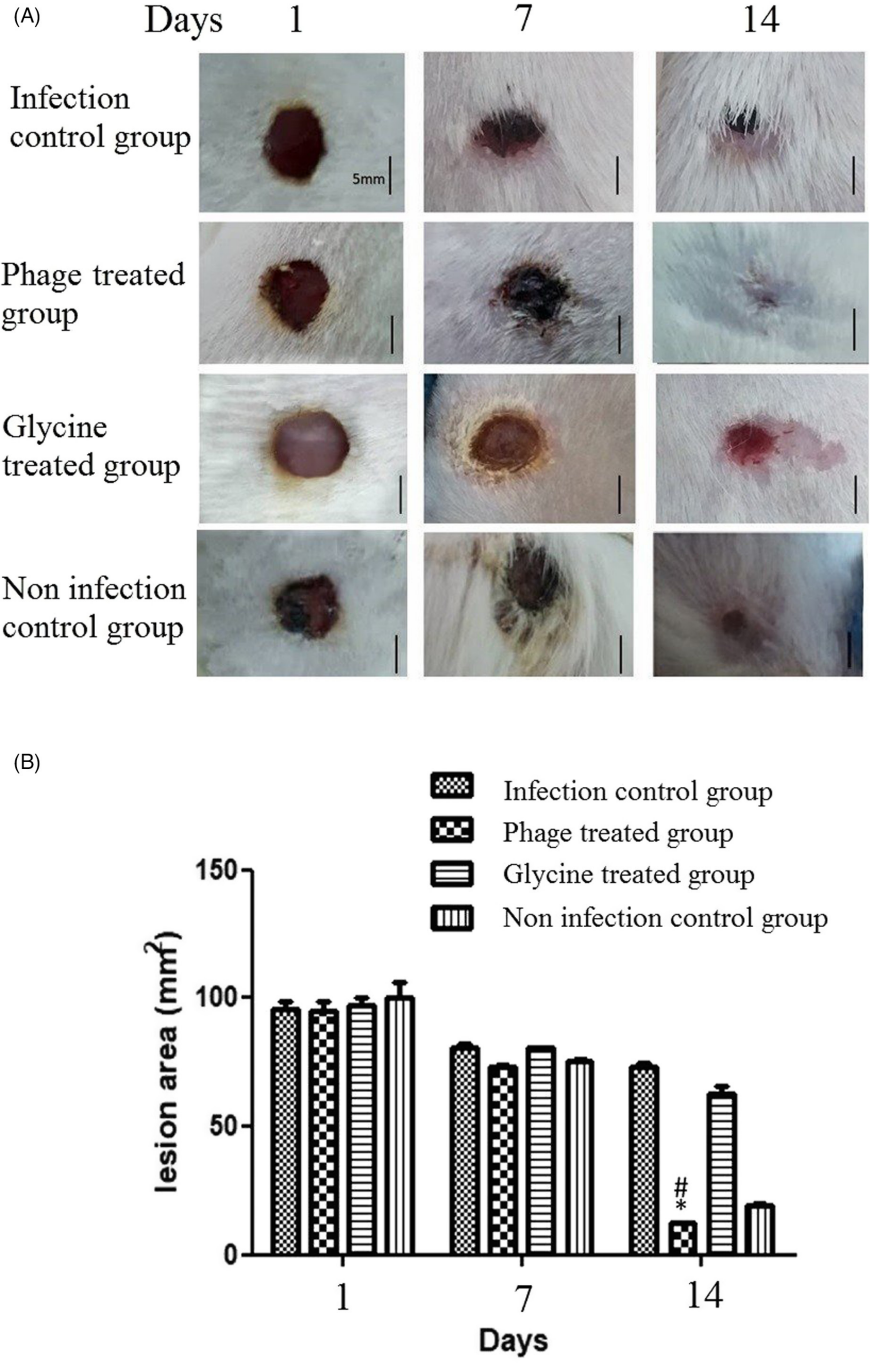
(A) Wound area in different treatment groups. Representative lesion images take for each group on day 0, 7, and 14 after infection induce. The phage uses at first day of infection, and on the 14th day after infection, (B) The mean size of the lesion area was significantly smaller in the phage‐treatment group as compared to the cases of the non‐treatment group (P < 0.05). Moreover, five rats were considered for each group. Scale bar: 5 mm. (n = 2 rat per day; *P < 0.05 versus phage treatment and# P < 0.05 versus non‐treatment). Statistical significance was measured by one‐way ANOVA. Data are presented as mean ± SD

The statistical analysis of the lesion area showed a significant difference between the phage‐treated infection lesion and non‐treated infection lesion over 14 days (*P* < 0.05) (Figure 6).

**FIGURE 7:**
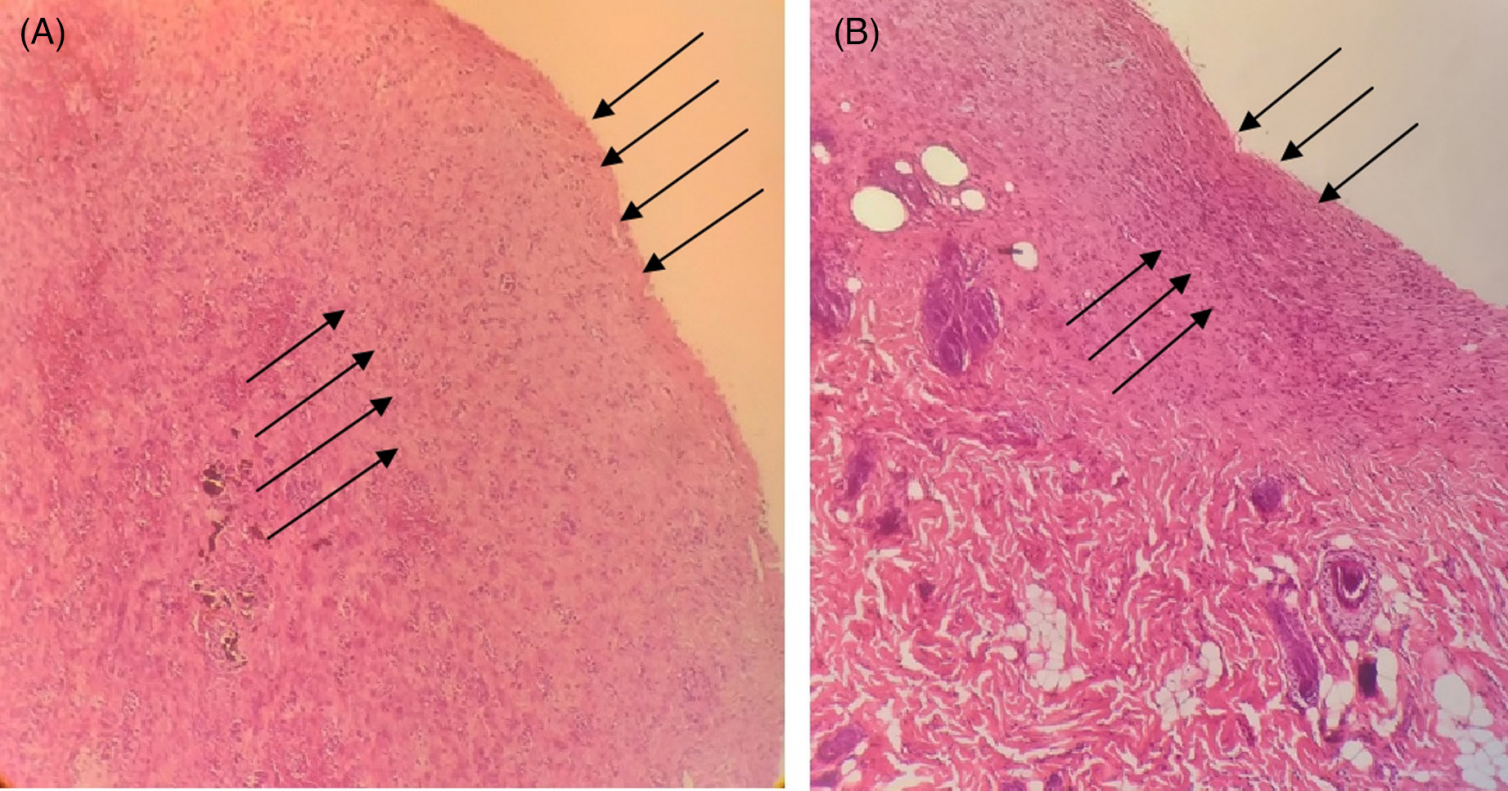
The epidermis and extracellular matrix in (A) nontreatment infection and (B) phage treated infection lesion Hematoxylin and Eosin staining (100X). Arrows shows the reepithelialization difference in A & B

**FIGURE 8:**
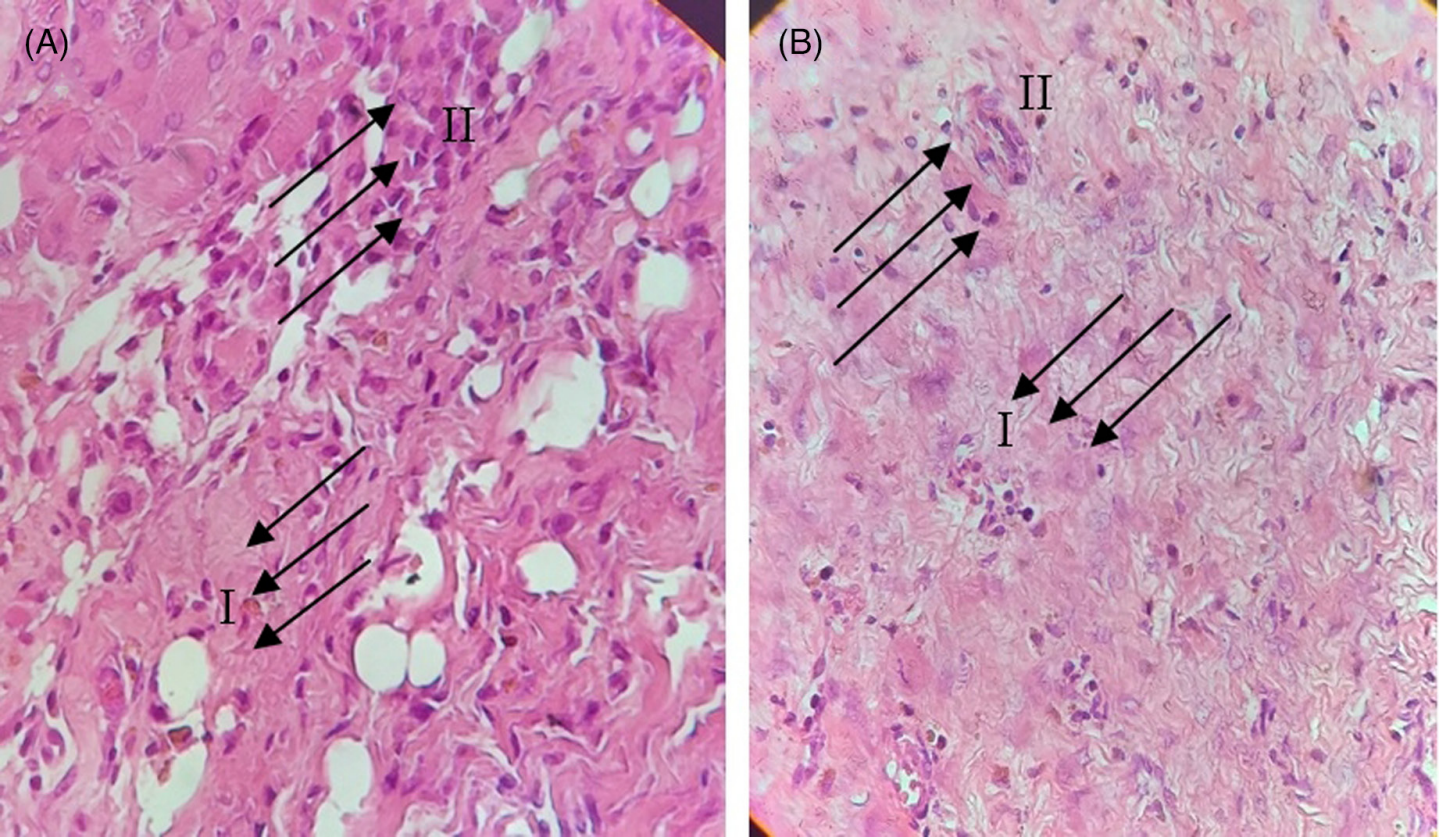
The infiltration and cell aggregation in (A) non treatment infection and (B) phage treated infection lesion Hematoxylin and Eosin staining (400X). Arrows in (I) show immune cell infiltration and (II) shows extra cellular matrix

In the histological analysis, it can be clearly seen that the epidermis and the extracellular matrix are thinner and slightly sparse in the non‐treated infectious lesion as compared to the case of the phage‐treated infectious lesion. This phenomenon causes new angiogenesis and reepithelization in non‐treated infectious rats (Figure 7). Analysis in 400× showed the cell infiltrates and aggregates in regenerative skin. The number of inflammatory cells in the phage‐treated rats had been reduced (Figure 8).

This data also showed that Inflammation existed in the non‐treated lesion area and the extracellular matrix (collagen and other proteins). All this evidence confirms that the phage had cleaned the bacteria and prevented the inflammation caused by bacterial infection during the physiological wound healing process.

## 4 DISCUSSION

*A. baumannii*, the most common clinically important gram‐negative MDR bacterium has dominated as a major causative agent of many problems for hospitals and patients, especially Intensive Care Unit (ICU) burn patients in recent years because of its fast spread in the hospital environment and its noticeable ability to quickly obtain resistance determinants against broad‐spectrum antimicrobial agents.^47^, ^48^

The results showed that all resistance genes tested in this study were identified as the high frequency in 19 isolates of XDR *A. baumannii*. Although all of the tested bacteria in this study were XDR, the emergence of resistance genes in some isolates indicated the ability of these isolates for transferring the resistance genes to sensitive isolates and increased antibiotic resistance in the microbial communities. These results are agreeing to WHO reports that infections associated with *A. baumannii* require new antibiotics.

According to Table 2, we also concluded that all of the tested bacteria in this study including gram‐positive and gram‐negative bacteria were not sensitive to this phage, indicating high host specificity. In other words, the vB‐AbauM‐Arak1 phage was specific to *A. baumannii*. So, this lytic vB‐AbauM‐Arak1 phage in contrast to antibiotics affecting normal flora has no lytic activity against other bacterial species, such as normal flora of the host body.

Using the single dosage of this phage against mouse model infection showed that the phage therapy in contrast to the antibiotic does not need repeated administrations, can successfully propagate in a single dosage, and completely eliminate the infectious bacterium. The microbial culture results also confirmed it. This lytic phage was effective in resolving wound infection caused by XDR *A. baumannii* in a rat model with a burn. It is a fact that the increasing use of antibiotics can potentially cause increasing the resistant bacterial strains to spread. So, applying the new alternative approaches could decrease the use of antibiotics, resulting in a gradual decrease in resistant bacteria. The most successful studies in the field of phage therapy have declared that bacteriophages probably can be used as an appropriate alternative to antibiotics.^49^, ^50^ As mentioned above, infection with antibiotic‐resistant bacteria could cause an increasing duration of hospital stays and health care costs.^51^, ^52^ Our results showed that phage‐treated rats recovered rapidly compared to other groups. Therefore, phage therapy could decrease the duration of hospital stays and health care costing.

The results from this study showing the anti‐inflammatory effect of phage are consistent with several prior studies.^31^, ^53^ As the findings showed, less skin inflammation was observed in the phage‐treated group. The prior studies showed that bacteriophages have anti‐inflammatory and immunoregulatory activities in addition to their antibacterial ability. In this case; more applications of phages would be expected (developed). The studies also showed that phages can increase the level of IL‐10 as an immunomodulatory.^31^, ^53^, ^54^

The therapeutic phage preparations should contain active phage particles in concentrations of 6.0 ± 7.0 log pfu/ml.^55^ In this study, we considered 7.0 log pfu/ml as an effective therapeutic titer (ETT) recommended as a therapeutic phage after incubation with the wound care products. Zhou et al.^56^ reported that the burst size and latent period of two *A. baumannii* specific phages, for example, WCHABP1 and WCHABP12 were 136 virions/infected cell, 10 min, and 175 virions/ infected cell, 20 min, respectively. Although the latent period time of the vB‐AbauM‐Arak1 phage (30 min) is more than WCHABP1 and WCHABP12 (10 and 20 min), its burst size (200 pfu/infected cell) is better than those phages. Therefore, the burst size and latent period of the lytic phage isolated in the current study could be suitable for phage therapy. According to our results, the persistence of vB‐AbauM‐Arak1 phage was about 100% and 80%, at the pH 7 and temperature of 40°C, respectively, indicating the lytic phage can survive in wound conditions and eliminate the *A. baumannii* infection due to optimum pH 7 and temperature of 37°C in humans. The results also demonstrated that the maximum adsorption (approximately 99%) of the vB‐AbauM‐Arak1 phage was observed after incubation of the mixture for 10 min. Previous studies reported several lytic phages against *A. baumannii* belonging to *Myoviridae* family. Leshkasheli et al.^57^ reported that the maximum attachment of vB_AbaM_3054 and vB_AbaM_3090 phages to the host cell was 89% ± 4% and 95% ± 2% after 10 min. In another study by Zhou et al.^56^ the phage adsorption rate of two WCHABP1 and WCHABP12 phages were determined as a high rate in which more than 99% were attached to the host after 10 min. Therefore, our data is in parallel with Zhou, et al.’s results and this shows that the vB‐AbauM‐Arak1 lytic phage exhibited very rapid adsorption to the bacterial host cell. This also draws attention to the use of this lytic phage toward developing an alternative choice for biocontrolling the *A. baumannii* infection.

As this study shows, one of the main applications of bacteriophages is preventing and biocontrolling of bacterial infections.^58^ According to its suitable stability in a wide range of pH and temperature, relatively short latent period, and appropriate burst size (approximately 200 phage particles per infected cell), vB‐AbauM‐Arak1could be the proper choice to prepare a phage cocktail against *A. baumannii* infections.

## 5 CONCLUSION

In conclusion, our study reports a new lytic phage against XDR *A. baumannii*, a natural, less harmful, and effective new approach to control infections. The isolated lytic phage will provide more open insight into treating XDR infections. More in vivo experiments and clinical trials are needed to prove the efficacy of the application of phage as an anti‐infective agent to treat XDR bacterial infections.

### 5.1 Limitations

The limitations of this study were that the identification of molecular characterization of bacteriophage whole genome and the plasmid profile antibiotic resistance genes could not be sequenced due to lack of experimental laboratory possibilities.

## Data Availability

Please contact corresponding author M.K. and first author E.GH. for data requests.
